# NSCLC mechanobiology: Delving into the intricate pathways involved in the dynamic interplay between tumors and the TME

**DOI:** 10.1038/s12276-024-01379-9

**Published:** 2025-01-01

**Authors:** Kostas A. Papavassiliou, Antonios N. Gargalionis, Efthimia K. Basdra, Athanasios G. Papavassiliou

**Affiliations:** 1 First University Department of Respiratory Medicine, ‘Sotiria’ Chest Hospital, Medical School, National and Kapodistrian University of Athens, Athens, Greece; 2 Laboratory of Clinical Biochemistry, ‘Attikon’ University General Hospital, Medical School, National and Kapodistrian University of Athens, Athens, Greece; 3 Department of Biological Chemistry, Medical School, National and Kapodistrian University of Athens, Athens, Greece

**Keywords:** Non-small-cell lung cancer, Oncogenesis

In the last few years, the cancer research community has increasingly acknowledged the contributions of cancer mechanobiology and the tumor microenvironment (TME) to the development and progression of different types of cancer^1^. Mounting evidence indicates that the mechanical properties of cancer cells and their interactions with immune cells, cancer-associated fibroblasts (CAFs), and extracellular matrix (ECM) components play pivotal roles in promoting the hallmarks of cancer^2^. Clinical trials investigating the mechanobiology-related features of tumors are ongoing, and cancer researchers are making substantial efforts to identify new therapeutic drug targets implicated in cancer mechanobiology. These steps have given rise to a novel molecular medicine field called cancer *mechanomedicine*. Researchers in this field aim to apply findings from studies on cancer mechanobiology to early cancer detection, diagnosis, and treatment^3^.

Non-small cell lung cancer (NSCLC) represents the majority of lung cancer cases worldwide, and therapeutic advances, including targeted agents and immune checkpoint inhibitors, have improved outcomes in patients with NSCLC. However, disease progression under current treatment regimens is common, and thus, the prognosis remains poor. Herein, we highlight the significance of NSCLC biomechanics in disease progression and the potential clinical benefit of therapeutically exploiting NSCLC mechanobiology.

Compared with normal lung parenchyma, lung cancer tissues exhibit significantly greater stiffness (~20–30 kPa)^4^. Similarly, NSCLC tumors display increased interstitial fluid pressure, which is the pressure developed by the body fluid within stromal tissue, and this has been reported to be associated with low recurrence-free survival^5^. These macroscopic mechanical properties of NSCLC are a result of mechanical alterations at the cellular and molecular levels. At the cellular level, CAFs and immune cells have been shown to participate in a dynamic interplay with cancer cells within the TME. The interaction of CAFs with NSCLC cells has been demonstrated to affect tumor progression. CAFs residing within the NSCLC TME are characterized by prominent heterogeneity and synthesize and secrete a plethora of factors, including ECM proteins, growth factors, and cytokines, which fuel cancer growth while maintaining an immunosuppressive environment and allowing cancer cells to invade, metastasize, and develop resistance to antitumor therapies^6^. Importantly, recent studies also suggest that CAF phenotypes are correlated with tumor aggressiveness, survival outcome, and treatment efficacy in NSCLC^7,8^.

At the molecular level, cancer and stromal cells sense and respond to aberrant mechanical cues from each other and from the ECM via a molecular process known as mechanosignaling (also termed mechanotransduction, i.e., conversion of a mechanical stimulus into a biochemical signal). Major factors involved in mechanosignaling include the proteins yes-associated protein (YAP) and transcriptional coactivator with PDZ-binding motif (TAZ), which function as transcriptional coactivators to induce gene expression programs in response to extracellular and intracellular mechanical stimuli. These gene expression programs endow NSCLC cancer cells with a number of fundamental malignant attributes^9^. For example, mechanically activated YAP/TAZ promotes the reprogramming of cancer cells into cancer stem cells. Additionally, the activation of YAP/TAZ via the promotion of mechanosignaling confers resistance to various targeted therapies, chemotherapeutic agents, and immune checkpoint inhibitors. YAP/TAZ activity also accelerates multiple steps in the metastatic process of cancer cells; this complex process is governed primarily by mechanical forces. Furthermore, YAP/TAZ function in TME-associated stromal cells to increase angiogenesis and generate/maintain CAFs, which, in turn, remodel the ECM and trigger the mechanoactivation of YAP/TAZ in cancer cells. Finally, YAP/TAZ mediate oncogenic crosstalk between immune cells and cancer cells, fostering immunosuppression. From a clinical perspective, these molecular mechanisms may explain why YAP/TAZ-induced transcriptional programs and elevated YAP/TAZ protein expression are correlated with a poor NSCLC prognosis^10^.

Targeting NSCLC mechanobiology appears to be an attractive therapeutic strategy that will address a key aspect of this heterogeneous lung malignancy. Treatments targeting NSCLC mechanobiology may include drugs that block the function of CAFs and their interplay with cancer cells, as well as agents that aim to inhibit YAP/TAZ mechanosignaling within cancer cells, CAFs, immune cells, and other stromal cells inhabiting the TME. To maximize the clinical benefit of these types of therapies, biomarker- or precision-guided approaches need to be developed and validated in relevant clinical trials. NSCLC mechanobiology seems to be a highly promising therapeutic target, and the cancer research community should continue exploring its complex pathways and underlying mechanisms to facilitate the development and translation of NSCLC mechanobiology-based therapeutics.
